# On evolutionary medicine and health disparities

**DOI:** 10.1093/emph/eoad008

**Published:** 2023-04-27

**Authors:** C Brandon Ogbunugafor, Fatimah Jackson

**Affiliations:** Department of Ecology and Evolutionary Biology, Yale University, New Haven, CT, USA; Public Health Modeling Unit, Yale School of Public Health, New Haven, CT, USA; Santa Fe Institute, Santa Fe, NM, USA; Department of Biology, College of Arts and Sciences, Howard University, Washington, DC, USA; Human Analytics Division, QuadGrid Research Laboratory in Bethesda, MD, USA

The mantra that ‘biology is an information science’, first popularized by Leroy Hood, is a commonly understood idea today [1]. The notion has aged well because of its prescience—it can be interpreted as having predicted that the next generation of biological questions would be defined by our ability to analyse, interpret and integrate data of various kinds.

Here we argue that health disparities—defined by the Centers for Disease Control as ‘preventable differences in the burden of disease, injury, violence, or opportunities to achieve optimal health that are experienced by socially disadvantaged populations [2]’—can similarly be defined as an information science. We suggest this because human experiences, culture and evolutionary and ecological forces are all critical dimensions to understanding how disparities arise and persist. We further emphasize this message through highlighting the special collection of articles in *The International Journal of Evolution, Medicine, and Public Health* entitled ‘Evolutionary Medicine and Health Disparities’. The contributions demonstrate the myriad of influences—evolutionary and ecological ones in particular—that impact the health of individuals and communities.

## THREE DIMENSIONS OF HEALTH DISPARITIES

Next, we outline three of the many drivers of differences in population health. As these ideas can be organized into many different categories, ours are not a definitive grouping and only serves to describe the multiplicity involved in understanding the complex problem of health disparities.

### Genetic variation

In 2023, the most cutting-edge questions in population genetics involve the genetic architecture of complex phenotypes, the source of phantom (or missing) heritability [3], the relevance of polygenic scores [4] and statistical methods for understanding the relationship between genotype and phenotype. This is especially true for traits with real-world consequences in *Homo sapiens*, such as behaviour and disease. When trying to understand health disparities, genetic information is an important dimension, as genetic variation can provide the informational scaffold upon which environments and contexts facilitate disparities manifest. This fact does not resemble genes ‘for’ any disparity, but rather, an important dimension that can play a role in how disease phenotypes manifest. Also, such a lens can incorporate modern debate around the use of race and ethnicity in studies in modern genomics [5] and medicine [6, 7]. This is necessary so that one does not fortify notions of biological race while in the process of studying how heritable genetic variation may contribute to population health.

### Evolutionary history

The notion that the forces of evolution—migration, selection, drift and mutation—neither play a role in why certain populations may acquire disease is hardly reductionist, nor does it reify differences between groups. We distinguish this from the ‘genetic variation’ category because understanding how evolutionary history influences disease requires very specific knowledge of the relationship between genes, environments and phenotype. That is, not all genetic underpinnings of the disease have a clear and tractable evolutionary (and adaptive) story.

This latter point is key and requires clarification. As with the genetic variation category described above, that forces of evolution are at work on specific geographical populations of humans does not imply that groups are essentially different. It only means that evolutionary history may have driven genetic variation associated with disease in various environmental contexts. These differences need not overlap with biological misunderstandings or social characterizations of racial groups. They simply highlight the complicated historical experience of *Homo sapiens* that has left signatures in genomes and contributes to how disease manifests.

### Socio-ecological context

This category contains the social, ecological and environmental factors which drive disease disparities. Understanding these involves a grounding in the social sciences, in order to frame the importance of social forces in biomedicine. This is an arena in which there is an extensive body of literature, led by scholars like Nancy Krieger, who have advanced an ecosocial theory to describe distributions of disease [8]. Others, like Thomas Leatherman and Alan Goodman, have offered a biocultural synthesis that attempts to integrate the political economy and other paradigms in biology [9]. And finally, public health experts like Felton Earls have even formally used the language of ecology in describing the web of social factors associated with well-being [10]. We should also note that disparities outlined with direct connections to structural issues are often framed as ‘health inequities’, which can be interpreted as a call to action to address fundamental causes of poor health outcomes in marginalized or disadvantaged groups.

We argue that in the context of health disparities, social and structural issues that underlie how the lives of *Homo sapiens* play out are paramount. For example, that racism has been a central actor in many areas of the American democratic project is uncontroversial to anyone operating on a fact-driven understanding of United States history. And these forces play a critical role in human development, health, sickness, diagnosis and treatment. Racism and other forces of discrimination are at work in many global settings and meaningfully contribute to health outcomes for populations around the world.

## HEALTH DISPARITIES IN LIGHT OF EVOLUTION (AND OTHER ACTORS)

Summarizing an evolutionary and ecological lens on health disparities need not suffer from the conceptual and empirical flaws of the adaptationist lens, whereby one attempts to over-explain worldly phenomena with simplistic evolutionary explanations [11]. That is, when we introduce evolutionary reasoning into the study of health disparities, we are not driven by a classical fallacy that there is necessarily a neat relationship between population differences in disease prevalence and adaptationist stories for how local populations evolve in response to a prior ecological challenge. Instead, evolutionary lenses acknowledge that disease disparities qualify as a complex system, composed of information of various kinds, and include forces such as structural violence, culture and policy.

This virtual issue of the *International Journal of Evolution, Medicine, and Public Health* features several manuscripts that address these different dimensions. These include several papers that highlight how early life exposures and/or socioeconomic status influences life trajectories [12–14]. The issue also features focused examinations of food insecurity during the COVID-19 pandemic and investigations into the causes of sleep disparities [15]. These studies—and the others which will be published and contribute to the special issue—represent the diversity of areas, approaches and insights that define the intersection between evolutionary medicine and health disparities, and the three areas outlined above (genetic variation, evolutionary history, socio-ecological context). Even more, there is an intersection between these topics and areas that the journal has covered in the past, including the relationship between the one health model and disparities [16]. Finally, the special issue highlights papers that reframe health disparities in terms of theoretical concepts from evolutionary theory such as niche construction, arguing that racism creates evolutionary mismatches through the existence of social structures that are maladaptive to individuals from certain communities, creating a context that foments unequal disease burden [17].

The forces that create health disparities act as emergent properties of the information described above. Consequently, the question of ‘how much’ of a health disparity is caused by any one of the categories is ill-posed: the genetic, evolutionary and social-ecological forces work in combination (perhaps *nonlinearly*) in crafting disparate health outcomes. We reiterate that these are not radical ideas, and are consistent with models of biology and disease, including the aforementioned ecosocial and biocultural models, and notions of an ‘exposome’, which refers to the movement to complement genomic approaches to health, with an understanding of how lifelong exposures to insults can contribute to disease [18].

Thedosious Dobzhansky’s famous quote that ‘nothing in biology makes sense except in light of evolution’ is among the most referenced in all of science and is even used as a motivator for evolutionary medicine: the field is driven by the idea that the forces of evolution underlie disease prevalence and dynamics. We argue that the same is true of health disparities, but with an important caveat: it is neither evolution alone nor any single kind of information that makes sense of these inequalities. Instead, sense only emerges from an intimate understanding of how these kinds of information interact.
